# Mortality trend due to Hepatitis B and C in the city of São Paulo, 2002–2016

**DOI:** 10.11606/s1518-8787.2020054002231

**Published:** 2020-11-23

**Authors:** Ana Paula Sayuri Sato, Inês Kazue Koizumi, Norma Suely de Oliveira Farias, Célia Regina Ciccolo da Silva, Maria Regina Alves Cardoso, Gerusa Maria Figueiredo

**Affiliations:** I Universidade de São Paulo Faculdade de Saúde Pública Departamento de Epidemiologia São PauloSP Brasil Universidade de São Paulo. Faculdade de Saúde Pública. Departamento de Epidemiologia. São Paulo, SP, Brasil; II Secretaria de Estado da Saúde de São Paulo Coordenadoria de Controle de Doenças Centro de Vigilância Epidemiológica “Prof. Alexandre Vranjac” São PauloSP Brasil Secretaria de Estado da Saúde de São Paulo. Coordenadoria de Controle de Doenças. Centro de Vigilância Epidemiológica “Prof. Alexandre Vranjac”. São Paulo, SP, Brasil; III Secretaria Municipal de Saúde de São Paulo Coordenadoria de Vigilância em Saúde Divisão de Vigilância Epidemiológica São PauloSP Brasil Secretaria Municipal de Saúde de São Paulo. Coordenadoria de Vigilância em Saúde. Divisão de Vigilância Epidemiológica. Programa Municipal de Hepatites Virais. São Paulo, SP, Brasil; IV Universidade de São Paulo Faculdade de Medicina Departamento de Medicina Preventiva São PauloSP Brasil Universidade de São Paulo. Faculdade de Medicina. Departamento de Medicina Preventiva. São Paulo, SP, Brasil

**Keywords:** Hepatitis B, Hepatitis C, Mortality, trends, Time Series Studies

## Abstract

**OBJECTIVE:**

To describe mortality due to hepatitis B and C as underlying cause in the municipality of São Paulo, verifying the trend of these rates, and to assess the association of these diseases with others, from 2002 to 2016.

**METHODS:**

This is a time series study on mortality due to hepatitis B and C according to sex, with data from the *Sistema de Informação de Mortalidade* (SIM – Mortality Information Sistem). Prais Winsten regression was used in rate trend analysis.

**RESULTS:**

The findings of this study showed a trend of decline of mortality from hepatitis B and C in recent years, particularly among males. These infections were important associated causes of liver cell carcinoma and HIV. The proportion of deaths under 70 years of age stands out.

**CONCLUSIONS:**

The study provides a baseline for research on mortality trend and the impact of interventions, given the history of expanded detection and supply of treatments, including the most recent antivirals in Brazil, since 2015.

## INTRODUCTION

Hepatitis B and C have great magnitude in the world, with estimates of about 257 million people chronically infected by the first (HBV) and 71 million by the second (HCV)^1^. In Brazil, a population survey performed in the Brazilian capitals and the Federal District in the 2010s showed prevalence of 0.37% and 7.40% for the surface antigen of virus B (HBsAg) and against its nucleus (Anti-HBc), respectively, and 1.38 for the antibody against virus C (Anti-HCV)^2^^–^^4^. This resulted in an estimate for the population inhabiting these areas of 430,658 of people with Anti-HCV reactivity, being 36.70% viremic^4^. In 2016, the Brazilian Ministry of Health estimated a prevalence of 0.70% nationwide and of 657,000 people with the active hepatitis C virus, aiming to establish a baseline to estimate the cases to be diagnosed and treated annually, in order to meet targets and eliminate viral hepatitis by 2030^5^. In a more recent publication, these estimates were updated to 0.53% and 632,000, respectively^6^. This information shows the importance of these infections in Brazil and the challenge for health services in detecting and treating patients.

Chronic HBV or HCV has been associated with an increased risk of death, especially from causes related to the development of liver diseases, such as cirrhosis or hepatocellular carcinoma (HCC). In 2015, 887,000 people died as a result of HBV infection^7^, and, in 2016, approximately 399,000 die each year with hepatitis C, mainly from cirrhosis and hepatocellular carcinoma^8^.

Viral hepatitis is among the main causes of death worldwide, being HCC the only cancer that substantially increased between 1990 and 2013^9^. In Brazil, the mortality rate from hepatitis B from 2000 to 2009 was 0.3/100,000 inhabitants-year^10^.

It should be noted that studies on hepatitis C mortality are scarce^5^. In the municipality of São Paulo, the mortality trend due to hepatitis B or C has not yet been evaluated. Thus, the objective of this study was to describe mortality due to hepatitis B and C as underlying cause in the city of São Paulo, verifying the trend of these rates, and to evaluate the participation of these infections as causes associated with others from 2002 to 2016.

## METHODS

This is an ecological time series on mortality from viral hepatitis B and C, from 2002 to 2016, including deaths of residents in the city of São Paulo.

The São Paulo capital has a territorial area of 1,522,986 km^2^, an estimated population of 11,638,802 inhabitants^11^ in 2016, and Municipal Human Development Index of 0.805^12^.

We used data on deaths in the municipality from 2002 to 2016, recorded in the *Sistema de Informações sobre Mortalidade* (SIM – Mortality Information System)^13^. The data source was the *Programa de Aprimoramento das Informações de Mortalidade* (PRO-AIM – Mortality Information Improvement Program), municipal administrator of the SIM and responsible for feeding information, sending it to the federal base and maintaining the municipal system. PRO-AIM has methods to improve the information contained in death certificates, in order to achieve and maintain a good quality of the system, such as sending letters to certifying doctors for clarification of doubts, investigation with the Instituto Médico Legal and the Serviço de Verificação de Óbitos, among others^14^. To estimate the rates, we used population estimates of the Fundação Sistema Estadual de Análise de Dados (SEADE)^11^.

Death from chronic hepatitis B was defined as those with the following codes from the International Classification of Diseases (ICD-10)^15^: B16.0 [acute hepatitis B with Delta-agent (co-infection) with hepatic coma], B16.1 [acute hepatitis B with Delta-agent (co-infection) without hepatic coma], B16.2 [acute hepatitis B without Delta-agent with hepatic coma], B16.9 [acute hepatitis B without Delta-agent and without hepatic coma], B17,0 [acute delta-super infection of hepatitis B carrier], B18.0 [chronic viral hepatitis B with Delta-agent] and B18.1 [chronic viral hepatitis B without Delta-agent] The definition of death from hepatitis C included ICD-10 codes B17.1 [acute hepatitis C] or B18.2 [chronic viral hepatitis C]^16^.

The underlying cause of death is defined by the World Health Organization as “(a) the disease or injury which initiated the train of morbid events leading directly to death, or (b) the circumstances of the accident or violence which produced the fatal injury.” Associated causes are all those described in the death certificate different from the underlying cause, that is, conditions that contributed to the death process (contributors) or that were complications of the underlying cause (consequential). All those described in the death certificate constitute multiple causes, that is, the underlying and associated causes^15^^,^^16^.

Initially, we only considered deaths with hepatitis B or C as underlying cause, to estimate the mortality rates and for trend analysis. Then, we analyzed the frequency with which hepatitis B or C were associated with deaths that had other underlying causes. For this, the associated causes for defining death were verified, that is, we considered the presence of the ICD-10 codes^15^ previously mentioned in any line of the death certificate^17^.

The gross mortality rate was estimated as the coefficient between total of deaths attributed to hepatitis B or C and the population in the studied age group residing in the municipality of São Paulo and multiplied by 100,000. The mortality coefficients were standardized by the direct method, using as reference the population of the 2010 census of the municipality of São Paulo.

The trend analysis was performed using a linear regression model for time series with the Prais-Winsten method, in order to minimize the first-order autocorrection of residues. The dependent variables were the standardized mortality rates from hepatitis B and C, and the independent variable was the calendar year^18^. We used the software Stata, version 15, for the statistical analysis of the data.

The project was approved by the Ethics Committee of the Municipal Health Secretariat of the city of São Paulo (Opinion No. 510.009, February 6, 2014).

## RESULTS

### Hepatitis B

From 2002 to 2016, 1,243 deaths were recorded in the SIM in the city of São Paulo mentioning viral hepatitis B, being 540 (43.44%) coded as underlying cause and 703 (56.55%) as associated cause.

Among the 540 deaths with hepatitis B as underlying cause, 127 (23.52%) were due to acute infection and 413 (76.48%) due to chronic infection. Among the patients, 69.60% were male and 30.40% were female. The predominant race/color was white (61.11%), and the majority (61.65%) had less than 8 years of schooling. The mean age of females was 66.00 years, higher than that of males (58.61 years). About two thirds of deaths were of people under 70 years of age, and this percentage is higher in males, with 75.83% (Table 1). No deaths of people under 15 years of age were recorded.

**Table 1:** Characteristics of deaths due to hepatitis B and C as underlying causes, São Paulo, SP, 2002–2016.

Variable		Hepatitis B (n = 540)		Hepatitis C (n = 3,194)	
		n	%	n	%
Sex					
	Female	164	30.40	1,433	44.90
	Male	376	69.60	1,761	55.10
Age group					
	15–34	20	3.71	45	1.30
	35–54	164	30.43	885	27.80
	50–69	191	35.43	1,372	43.00
	≥ 70	165	30.43	892	27.90
Race/color					
	White	330	61.11	2,428	76.02
	Black	33	6.11	137	4.29
	Brown	105	19.44	400	12.52
	Yellow	29	5.37	56	1.75
	Indigenous	–	–	1	0.03
	Ignored	43	7.96	172	5.39
Schooling					
	None	35	6.48	169	5.29
	1–3	104	19.26	652	20.41
	4–7	115	21.30	741	23.20
	8–11	99	18.33	657	20.57
	≥ 12	59	10.93	416	13.02
	Ignored	128	23.70	559	17.50

As for the associated causes, we found 289 (53.52%) deaths with liver cirrhosis and 22 (4.07%) with hepatocellular carcinoma (data not shown in tables).

**Figure 1:**
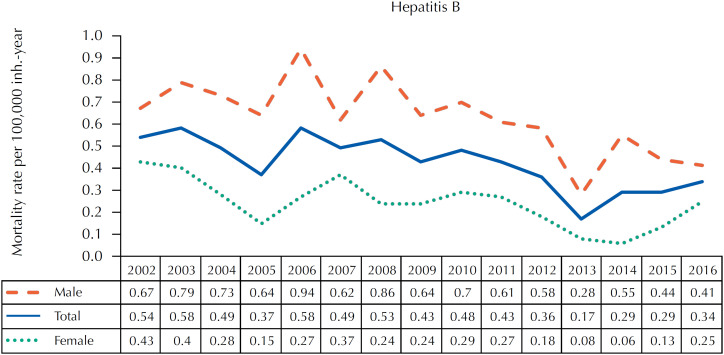
Standardized mortality rates for hepatitis B as underlying cause, according to sex and year of death, São Paulo, SP, 2002–2016.

Source: SIM/PRO-AIM/CEINFO/SMSSP.

From 2002 to 2016, mortality rates from hepatitis B as underlying cause varied from 0.17 (2003 and 2006) to 0.58 (2013) per 100,000 inhabitants-year. There was an annual decline of 5.10% (95%CI: −8.26; −1.82%) in males (-4.75%; 95%CI: −7.03; −2.42%), but not in females (-6.79%; 95%CI: −14.10; 1.14%) (Figure 1 and Table 2).

**Table 2:** Mean annual variation in mortality rates from hepatitis B and C as underlying causes, according to sex and study period, São Paulo, 2002–2016.

Type/period	Sex	Mean annual variation (%)	95% confidence interval	
			-	+
**Hepatitis B**				
2002–2016	General	-5.10	-8.26	-1.82
	Male	-4.75	-7.03	-2.42
	Female	-6.79	-14.10	1.14
**Hepatitis C**				
2002–2009	General	5.65	2.99	8.39
	Male	4.60	2.41	6.84
	Female	6.90	3.03	10.91
2009–2016	General	-4.79	-7.44	-2.07
	Male	-4.95	-7.63	-2.19
	Female	-5.29	-10.98	0.76

Hepatitis B was mentioned in 703 deaths whose underlying cause were diseases constant in ICD-10 chapters. The highest proportion is associated with diseases from Chapter II, referring to neoplasms (C00-D48), with 42.53% of deaths (n = 299), and Chapter I, referring to infectious and parasitic diseases (A00-B99), with 30.44% (n = 214). Among neoplasms, liver cell carcinoma (C22.0) (56.18%, 169/299) and unspecified malignant liver neoplasm (C22.9) stand out, with 57 deaths (19.06%, 57/299). Among infectious diseases, those associated with HIV stand out (B20.0, B20.1, B20.3, B20.6, B20.7, B20.8, B21.0, B22.7, B24), with 172 deaths (80.37%, 172/214). Among these, 14 deaths also stand out, in which the underlying cause was hepatitis C (B17.1 and B18.2) (6.54%, 14/214). In Chapter XI – Diseases of the Digestive System, 28 deaths due to diseases related to alcohol intake are noticeable (K70.0, K70.1, K70.3, K70.4 and K70.9) (data not shown in tables).

### Hepatitis C

From 2002 to 2016, 6,419 deaths were recorded with mention to hepatitis C, being 3,194 (49.76%) as underlying cause and 3,225 (50.24%) as an associated cause in residents of the city of São Paulo.

Among the 3,194 people who died with hepatitis C as underlying cause, 55.10% were males and 44.90% females. The race/color white was in 76.02% of the records, and 48,90% of the patients had less than 8 years of schooling. Mean age was 61.72 years, greater among women (65.64 years) compared with men (58.51 years). There were no records of deaths in children under 15 years of age. It was found that 72.12% of the deaths were of people under 70 years of age, being this percentage higher in males, with 81.91% (Table 1).

In addition, 1,801 deaths (56.41%) from hepatitis C also had hepatic cirrhosis as associated cause. Of these cases, women (44.50%) had a mean age of 66.22 years, and men (55.50%), 57.93 years. We identified 196 (6,14%) deaths with hepatocellular carcinoma as associated cause (data not shown in tables).

**Figure 2:**
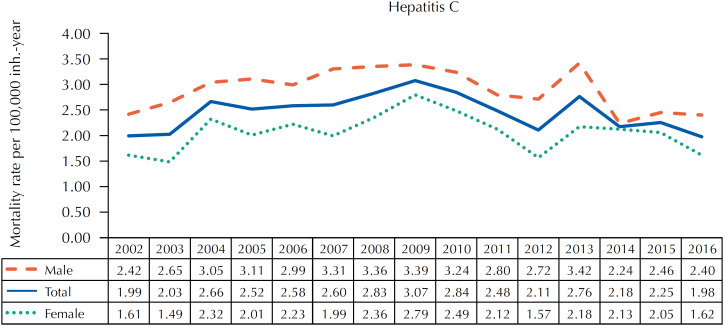
Standardized mortality rates for hepatitis C as underlying cause, according to sex and year of death, São Paulo, SP, 2002–2016.

Source: SIM/PRO-AIM/CEINFO/SMSSP.

The mortality rates from hepatitis C as underlying cause showed an increase of 5.65% per year (95%CI 2.99; 8.39%) between 2002 (1.99/100,000 inhabitants-year) and 2009 (3.07/100,000 inhabitants-year), both in males (4.60%; 95%CI 2.41;6.84%) and females (6.90%; 95%CI 3.03;10.91%). From 2009 to 2016, there was an annual decrease trend of 4.79% (95%CI −7.44; −2.07), decreasing to 1.98/100,000 inhabitants in 2016. This decline was found in males (-4.95%; 95%CI −7.63; −2.19%), but not in females (-5.29%; 95%CI −10.98; 0.76%) (Figure 2 and Table 2).

In relation to the 3,225 deaths mentioning hepatitis C as associated cause, the greater proportion has neoplasms as underlying cause (Chapter II of ICD-10, C00-D48, n = 1,246, 38.63%), followed by infectious and parasitic diseases (Chapter I of ICD-10, A00-B99, n = 893, 27.69%). Among the neoplasms, liver cell carcinoma (C22.0) accounted for 59.95% of deaths (747/1,246), followed by unspecified malignant liver neoplasm (C22.9) (14.77%, 184/1,246). Among infectious diseases, 78.61% of deaths (702/893) had diseases associated to HIV as underlying cause (B20.0, B20.1, B20.2, B20.3, B20.6, B20.7, B20.8, B21.0, B21.1, B21.7, B21.8, B22.0, B22.7, B23.2, B24), and 7.61% (68/893) had hepatitis B (B16.2, B16.9, B18.1). It is worth noting that 113 individuals died of liver diseases related to alcohol intake (K70.0, K70.1, K70.3, K70.4 and K70.9) (data not shown in tables).

## DISCUSSION

The findings of this study showed a trend of declined mortality from hepatitis B and C in recent years in the municipality of São Paulo, particularly in males. These infections were important causes associated to liver cell carcinoma and HIV. In addition, the proportion of individuals who died under 70 years of age stands out (about 70%).

The analysis of mortality trend from hepatitis B showed reduction, particularly in men. In the USA, the mortality trend from hepatitis B between 1999 and 2007 showed that the annual age-adjusted mean was relatively constant^19^. But, in other places, such as Taiwan, a hyper-endemic region in past decades, there has been sharp decreases^4^^,^^20^^,^^21^. We suggest that the decreased mortality rates from hepatitis B in Brazil may be attributed in part to vaccination, with decreased prevalence, but also to prevention measures against infections by HIV initiated in the 1980s, as observed in other countries^4^^,^^22^^,^^23^.

In relation to mortality from hepatitis C, there was a mean increase trend of 5.65% per year between 2002 and 2009, in both sexes, and reduction of 4.79% from 2009 to 2016, in males. In the US, the standardized annual mortality rate increased and exceeded AIDS mortality between 1999 and 2007^19^. In Brazil, the percentage drop in the second period stands out, which may be, in part, due to the treatment instituted in the Country at the beginning of the previous decade, with double therapy, used until 2014, including pegylated interferon as one of its components, in addition to the increased uptake of infected people by health services^24^^–^^26^. In 2015, the Ministry of Health instituted the treatment with new direct-acting antivirals, which show efficacy of more than 90% in curing hepatitis C and lower rates of adverse effects^27^.

Clinical care for the patient with HCV has advanced considerably, due to the evolution of diagnostic procedures and the improvement in therapy and prevention. The treatment of the infection has been proposed and improved for about 20 years, with a therapeutic arsenal of progressively better efficacy.

In many countries, the incidence of hepatitis B and C has decreased due to fewer new infections. However, the prevalence of severe liver diseases continues to increase, since there is a contingent of people infected with HBV and HCV in past decades who are unaware of their condition or who were diagnosed late, when they show signs and symptoms of severe liver disease, such as cirrhosis and HCC, which contributes to premature death^9^^,^^28^.

In the US, a cohort study, from 2006 to 2010, showed mean age of 57 years for deaths from hepatitis C^29^. Another study conducted in the city of New York, with a casuistry from 2000 to 2011, pointed out that 64.10% of the deaths of individuals infected by HCV occurred before 65 years of age^30^.

In this study, it was observed that at least two thirds of deaths from hepatitis B and C affected individuals under the age of 70, that is, those born between 1945 and 1965 (“baby boomers”). In the case of hepatitis C, these people became infected mainly before the incorporation of screening in blood banks and new technologies of products and processes related to the control of blood and blood products, as well as biosecurity standards in health services^31^. This finding strengthens the recommendation for Anti-HCV testing in people born in this period, since the infection may be asymptomatic in 75% of the cases^32^, as well as for instituting early treatment and vaccination against HBV in these carriers, according to the recommended protocol, preventing the aggravation of their condition^33^.

The significant proportion of hepatitis B and C contributing as causes associated with HCC and HIV is consistent in other findings in literature. A systematic review of studies published in several countries showed that chronic viral hepatitis due to HBV and HCV contribute to the majority of HCC occurences^33^. Globally, 78% of HCC infections were attributed to HBV (53%) and HCV (25%), confirming that chronic hepatitis B and C represent the main cause of primary liver cancer in the world^7^^–^^8^^,^^33^. The co-infection of viral hepatitis with HIV may be explained by the common routes of infection, mainly parenteral and sexual^34^, and is associated with a higher risk of death^27^^,^^35^. Progression from liver fibrosis to cirrhosis is accelerated in individuals with concomitant hepatitis C and HIV^36^.

Mortality data from this study may be underestimated due to the absence of hepatitis B and C in the death certificate, either as underlying or associated cause^28^. The associated causes of death were also studied to try to understand the effect of the under-registration of hepatitis B and C as underlying cause, especially when associated with liver cirrhosis, HCC and HIV infection^37^.

Mortality statistics according to underlying cause no longer show the total impact of a disease or aggravation in a set of deaths^38^. This study enabled this broader and more complete view, evidencing the presence of viral hepatitis B and C as associated causes, in order to contribute to the knowledge of the epidemiological profile of these diseases in our environment.

The data found show the importance of prevention and early diagnosis and treatment of hepatitis B and C. The contribution of this study is to provide a baseline for new studies on mortality trend and evaluation of the impact of new interventions, considering the expansion of diagnosis and treatment offer, including the most recent antivirals in Brazil, since 2015^27^, regardless of the degree of liver fibrosis, since 2019^39^. In addition, these results may support the achievement of predicted goals for eliminating hepatitis B and C in the city of São Paulo^39^^,^^40^.
