# Supplementary material for: Mortality trend due to Hepatitis B and C in the city of São Paulo, 2002–2016
Source: Rev Saude Publica. 2020 Nov 23;54:124. doi: 10.11606/s1518-8787.2020054002231 (PMC7688257; doi:10.11606/s1518-8787.2020054002231)
Supplement: Translation

# Translation: Tendência de mortalidade por hepatites B e C no município de São Paulo, 2002–2016

**Authors:** Ana Paula Sayuri Sato, Inês Kazue Koizumi, Norma Suely de Oliveira Farias, Célia Regina Ciccolo da Silva, Maria Regina Alves Cardoso, Gerusa Maria Figueiredo

**Affiliations:** I São PauloSP Brasil Universidade de São Paulo. Faculdade de Saúde Pública. Departamento de Epidemiologia. São Paulo, SP, Brasil; II São PauloSP Brasil Secretaria de Estado da Saúde de São Paulo. Coordenadoria de Controle de Doenças. Centro de Vigilância Epidemiológica “Prof. Alexandre Vranjac”. São Paulo, SP, Brasil; III São PauloSP Brasil Secretaria Municipal de Saúde de São Paulo. Coordenadoria de Vigilância em Saúde. Divisão de Vigilância Epidemiológica. Programa Municipal de Hepatites Virais. São Paulo, SP, Brasil; IV São PauloSP Brasil Universidade de São Paulo. Faculdade de Medicina. Departamento de Medicina Preventiva. São Paulo, SP, Brasil

**Keywords:** Hepatite B, Hepatite C, Mortalidade, tendências, Estudos de Séries Temporais

## Abstract

**OBJETIVO:**

Descrever a mortalidade por hepatites B e C como causa básica no município de São Paulo, verificando a tendência dessas taxas, e avaliar a associação dessas doenças a outras no período de 2002 a 2016.

**MÉTODOS:**

Trata-se de um estudo de série temporal sobre mortalidade por hepatites B e C segundo sexo, com dados do Sistema de Informação de Mortalidade. A regressão de Prais Winsten foi usada na análise de tendência das taxas.

**RESULTADOS:**

Os achados do presente estudo mostraram tendência de declínio da mortalidade por hepatites B e C nos últimos anos, particularmente entre pessoas do sexo masculino. Essas infecções foram causas associadas importantes ao carcinoma de células hepáticas e ao HIV. Destaca-se a proporção de óbitos com menos de 70 anos de idade.

**CONCLUSÕES:**

O estudo fornece uma linha de base para pesquisas de tendência de mortalidade e de impacto de intervenções, visto o histórico de ampliação da detecção e oferta de tratamentos, incluindo os mais recentes antivirais no Brasil, desde 2015.

## INTRODUÇÃO

As hepatites B e C apresentam grande magnitude no mundo, com estimativas de cerca de 257 milhões de pessoas cronicamente infectadas pelo vírus da primeira (VHB) e 71 milhões pelo vírus da segunda (VHC)^1^. No Brasil, um inquérito populacional realizado nas capitais e no Distrito Federal na década de 2010, com pessoas de 10 a 69 anos, apresentou prevalência de 0,37% e 7,40% para o antígeno de superfície do vírus B (HBsAg) e contra o núcleo (Anti-HBc), respectivamente, e de 1,38% para o anticorpo contra o vírus C (Anti-HCV)^2^^–^^4^. Isso resultou em uma estimativa para população residente nessas áreas de 430.658 pessoas Anti-HCV reagentes, sendo 36,70% virêmicas^4^. Em 2016, o Ministério da Saúde estimou prevalência de 0,70% no país todo e de 657.000 pessoas com o vírus ativo da hepatite C, visando estabelecer uma linha de base para calcular os casos a diagnosticar e tratar anualmente, a fim de cumprir metas e eliminar as hepatites virais até 2030^5^. Em publicação mais recente essas estimativas foram atualizadas para 0,53% e 632.000, respectivamente^6^. Essas informações mostram a importância dessas infecções no Brasil e o desafio para os serviços de saúde em detectar e tratar os portadores.

A hepatite crônica pelo VHB ou VHC tem sido associada ao aumento do risco de morte, sobretudo por causas relacionadas ao desenvolvimento de doenças hepáticas, como cirrose ou hepatocarcinoma (HCC). Em 2015, 887.000 pessoas morreram em consequência da infecção pelo VHB^7^, e em 2016, aproximadamente 399.000 morrem a cada ano com hepatite C, principalmente por cirrose e carcinoma hepatocelular^8^.

As hepatites virais estão entre as principais causas de morte em todo o mundo, sendo o HCC o único câncer que aumentou substancialmente entre os anos 1990 e 2013^9^. No Brasil, a taxa de mortalidade por hepatite B no período de 2000 a 2009 foi de 0,3/100.000 habitantes-ano^10^.

Ressalte-se que os estudos sobre mortalidade por hepatite C no país são escassos^5^. No município de São Paulo, a tendência de mortalidade por hepatite B ou C não foi avaliada até o momento. Assim, o objetivo deste estudo é descrever a mortalidade por hepatites B e C como causa básica no município de São Paulo, verificando a tendência dessas taxas, e avaliar a participação dessas infecções como causas associadas a outras no período de 2002 a 2016.

## MÉTODOS

Trata-se de estudo ecológico de séries temporais sobre a mortalidade por hepatites virais B e C, no período de 2002 a 2016, compreendendo óbitos de residentes no município de São Paulo.

A capital paulista tem área territorial de 1.522,986 km^2^, população estimada em 11.638.802 habitantes^11^ para 2016 e Índice de Desenvolvimento Humano Municipal de 0,805^12^.

Utilizaram-se dados referentes aos óbitos no município ocorridos no período de 2002 a 2016, registrados no Sistema de Informações sobre Mortalidade (SIM)^13^. A fonte de dados foi o Programa de Aprimoramento das Informações de Mortalidade (PRO-AIM), gestor municipal do SIM e responsável por alimentar as informações, enviá-las à base federal e fazer a manutenção do sistema municipal. O PRO-AIM possui métodos para aprimorar as informações constantes nas declarações de óbito, com intuito de alcançar e manter a boa qualidade do sistema, como o envio de cartas aos médicos atestantes para o esclarecimento de dúvidas, investigação junto ao Instituto Médico Legal e ao Serviço de Verificação de Óbitos, entre outros^14^. Para o cálculo das taxas, foram utilizadas estimativas populacionais da Fundação Sistema Estadual de Análise de Dados (Seade)^11^.

Definiram-se como óbitos por hepatite B aqueles que apresentavam os seguintes códigos da Classificação Internacional de Doenças (CID-10)^15^: B16.0 [hepatite aguda B com agente Delta (coinfecção) com coma hepático], B16.1 [hepatite aguda B com agente Delta (coinfecção) sem coma hepático], B16.2 (hepatite aguda B sem agente Delta, com coma hepático), B16.9 (hepatite aguda B sem agente Delta, sem coma hepático), B17.0 (superinfecção Delta aguda de portador de hepatite crônica B), B18.0 (hepatite viral crônica B com agente Delta) e B18.1 (hepatite viral crônica B sem agente Delta). A definição de óbito por hepatite C compreendeu os códigos B17.1 (hepatite C aguda) ou B18.2 (hepatite C crônica) da CID-10^15^.

A causa básica de morte é definida pela Organização Mundial da Saúde como “(a) a doença ou lesão que iniciou a sucessão de eventos patológicos que levou diretamente à morte, ou (b) as circunstâncias do acidente ou violência que produziu a lesão fatal”. Causas associadas são todas as descritas na declaração de óbito diferentes da causa básica, ou seja, condições que contribuíram no processo de falecimento (contribuintes) ou que foram complicações da causa básica (consequenciais). Constituem causas múltiplas todas aquelas descritas na declaração de óbito, ou seja, as causas básicas e as associadas^15^^,^^16^.

Inicialmente consideraram-se apenas os óbitos cuja causa básica foi a hepatite B ou C, para o cálculo das taxas de mortalidade e para a análise de tendência. Em seguida, foi analisada a frequência com que as hepatites B e C estavam associadas a óbitos que tinham outra causa básica. Para isso, verificaram-se as causas associadas para definição de morte, ou seja, considerou-se a presença dos códigos da CID-10^15^ citados anteriormente em qualquer linha da declaração de óbito^17^.

A taxa bruta de mortalidade foi calculada como o quociente entre o total de óbitos atribuídos à hepatite B ou C e a população na faixa etária estudada residente no município de São Paulo, em cada ano e multiplicado por 100.000. A padronização dos coeficientes de mortalidade foi realizada pelo método direto, utilizando como referência a população do censo de 2010 do município de São Paulo.

A análise de tendência foi realizada por meio de modelo de regressão linear para séries temporais com método de Prais-Winsten, a fim de minimizar a autocorrelação de primeira ordem dos resíduos. As variáveis dependentes foram as taxas padronizadas de mortalidade por hepatite B e C, e a variável independente foi o ano-calendário^18^. Utilizou-se o *software* Stata versão 15 para a análise estatística dos dados.

O projeto foi aprovado pelo Comitê de Ética da Secretaria Municipal de Saúde da Prefeitura da Cidade de São Paulo (Parecer n^o^ 510.009, de 6 de fevereiro de 2014).

## RESULTADOS

### Hepatite B

No período de 2002 a 2016, foram registrados no Sistema de Informação sobre Mortalidade do município de São Paulo 1.243 óbitos com menção à hepatite viral B, sendo 540 (43,44%) codificados como causa básica e 703 (56,55%) como causa associada.

Entre os 540 óbitos com hepatite B como causa básica, 127 (23,52%) foram decorrentes de infecção aguda e 413 (76,48%), crônica. Dentre os pacientes, 69,60% eram do sexo masculino e 30,40%, do feminino. A raça/cor branca foi predominante (61,11%), e a maioria (61,65%) tinha menos de 8 anos de escolaridade. A média da idade do sexo feminino foi de 66,00 anos, maior do que a do sexo masculino (58,61 anos). Cerca de dois terços dos óbitos foram de pessoas com menos de 70 anos de idade, e esta porcentagem é maior no sexo masculino, com 75,83% (Tabela 1). Não foram registrados óbitos de pessoas abaixo de 15 anos de idade.

**Tabela 1:** Características dos óbitos por hepatites B e C como causas básicas, São Paulo, SP, 2002–2016.

Variável		Hepatite B (n = 540)		Hepatite C (n = 3194)	
		n	%	n	%
Sexo					
	Feminino	164	30,40	1433	44,90
	Masculino	376	69,60	1761	55,10
Faixa etária					
	15–34	20	3,71	45	1,30
	35–54	164	30,43	885	27,80
	55–69	191	35,43	1372	43,00
	≥ 70	165	30,43	892	27,90
Raça/cor					
	Branca	330	61,11	2428	76,02
	Preta	33	6,11	137	4,29
	Parda	105	19,44	400	12,52
	Amarela	29	5,37	56	1,75
	Indígena	–	–	1	0,03
	Ignorada	43	7,96	172	5,39
Escolaridade					
	Nenhuma	35	6,48	169	5,29
	1–3	104	19,26	652	20,41
	4–7	115	21,30	741	23,20
	8–11	99	18,33	657	20,57
	≥ 12	59	10,93	416	13,02
	Ignorada	128	23,70	559	17,50

Quanto às causas associadas, foram encontrados 289 (53,52%) óbitos com cirrose hepática, e 22 (4,07%) com carcinoma hepatocelular (dados não apresentados em tabelas).

No período de 2002 a 2016, as taxas de mortalidade por hepatite B como causa básica variaram de 0,17 (2003 e 2006) a 0,58 (2013) por 100.000 habitantes-ano. Houve declínio anual de 5,10% (IC95%: −8,26; −1,82%) em pessoas do sexo masculino (-4,75%; IC95%: −7,03; −2,42%), mas não nas do feminino (-6,79%; IC95%: −14,10; 1,14%) (Figura 1 e Tabela 2).

**Figura 1:** Taxas de mortalidade padronizadas por hepatite B como causa básica, segundo sexo e ano do óbito, São Paulo, SP, 2002–2016.

Fonte: SIM/PRO-AIM/CEINFO/SMSSP.

**Tabela 2:** Variação anual média das taxas de mortalidade por hepatites B e C como causas básicas, segundo sexo e período estudado, São Paulo, SP, 2002-2016.

Tipo/período	Sexo	Variação anual média (%)	Intervalo de confiança de 95%	
			-	+
**Hepatite B**				
2002–2016	Geral	-5,10	-8,26	-1,82
	Masculino	-4,75	-7,03	-2,42
	Feminino	-6,79	-14,10	1,14
**Hepatite C**				
2002–2009	Geral	5,65	2,99	8,39
	Masculino	4,60	2,41	6,84
	Feminino	6,90	3,03	10,91
2009–2016	Geral	-4,79	-7,44	-2,07
	Masculino	-4,95	-7,63	-2,19
	Feminino	-5,29	-10,98	0,76

Encontrou-se menção à hepatite B em 703 óbitos cuja causa básica foram doenças constantes em capítulos da CID-10. A maior proporção está associada a doenças do Capítulo II, referente a neoplasias (C00-D48), com 42,53% dos óbitos (n = 299), e ao Capítulo I, referente a doenças infecciosas e parasitárias (A00-B99), com 30,44% (n = 214). Entre as neoplasias destacam-se o carcinoma de células hepáticas (C22.0) (56,18%, 168/299) e a neoplasia maligna do fígado não especificada (C22.9), com 57 óbitos (19,06%, 57/299). Entre as doenças infecciosas sobressaem-se as associadas ao HIV (B20.0, B20.1, B20.3, B20.6, B20.7, B20.8, B21.0, B22.7, B24), com 172 óbitos (80,37%, 172/214). Dentre essas também se destacam 14 falecimentos cuja causa básica foi hepatite C (B17.1 e B18.2), (6,54%, 14/214). No Capítulo XI – Doenças do Aparelho Digestivo, chamam atenção 28 óbitos por doenças relacionadas à ingestão de álcool (K70.0, K70.1, K70.3, K70.4 e K70.9) (dados não apresentados em tabelas).

### Hepatite C

No período de 2002 a 2016 foram registrados 6.419 óbitos com menção à hepatite C, sendo 3.194 (49,76%) como causa básica e 3.225 (50,24%) como causa associada em residentes no município de São Paulo.

Entre as 3.194 pessoas falecidas com hepatite C como causa básica, 55,10% eram do sexo masculino e 44,90% do feminino. A raça/cor branca constava em 76,02% dos registros, e 48,90% dos pacientes tinham menos de 8 anos de escolaridade. A média de idade foi de 61,72 anos, maior entre as mulheres (65,64 anos) em comparação aos homens (58,51 anos). Não foram registrados óbitos em menores de 15 anos de idade. Constatou-se que 72,12% dos óbitos foram de pessoas com menos de 70 anos de idade, sendo esta porcentagem maior no sexo masculino, com 81,91 % (Tabela 1).

Além disso, 1.801 óbitos (56,41%) por hepatite C tinham cirrose hepática como causa associada. Destes casos, as mulheres (44,50%) apresentaram média de idade de 66,22 anos e os homens (55,50%), 57,93 anos. Identificaram-se 196 (6,14%) óbitos com carcinoma hepatocelular como causa associada (dados não apresentados em tabelas).

As taxas de mortalidade por hepatite C como causa básica apresentaram tendência de aumento de 5,65% ao ano (IC95% 2,99;8,39%) entre 2002 (1,99/100.000 habitantes-ano) e 2009 (3,07/100.000 habitantes-ano), tanto no sexo masculino (4,60%; IC95% 2,41;6,84%) quanto no feminino (6,90%; IC95% 3,03;10,91%). De 2009-2016, houve tendência de diminuição anual de 4,79% (IC95% −7,44; −2,07), passando para 1,98/100.000 habitantes em 2016. Esse declínio esteve presente em indivíduos do sexo masculino (-4,95%; IC95% −7,63; −2,19%), mas não do feminino (-5,29%; IC95% −10,98; 0,76%) (Figura 2 e Tabela 2).

**Figura 2:** Taxas de mortalidade padronizadas por hepatite C como causa básica, segundo sexo e ano do óbito, São Paulo, SP, 2002–2016.

Fonte: SIM/PRO-AIM/CEINFO/SMSSP.

Em relação aos 3.225 óbitos com menção à hepatite pelo vírus C como causa associada, a maior proporção tinha como causa básica as neoplasias (Capítulo II do CID-10, C00-D48, n = 1.246, 38,63%), seguidas de doenças infecciosas e parasitárias (Capítulo I do CID-10, A00-B99, n = 893, 27,69%). Entre as neoplasias, o carcinoma de células hepáticas (C22.0) correspondeu a 59,95% dos óbitos (747/1.246), seguido de neoplasia maligna do fígado não especificada (C22.9) (14,77%, 184/1.246). Entre as doenças infecciosas, 78,61% dos óbitos (702/893) tinham doenças associadas ao HIV como causa básica (B20.0, B20.1, B20.2, B20.3, B20.6, B20.7, B20.8, B21.0, B21.1, B21.7, B21.8, B22.0, B22.7, B23.2, B24), e 7,61% (68/893) tinham a hepatite B (B16.2, B16.9, B18.1). Destaca-se que 113 indivíduos faleceram por doenças hepáticas relacionadas à ingestão de álcool (K70.0, K70.1, K70.3, K70.4 e K70.9) (dados não apresentados em tabelas).

## DISCUSSÃO

Os achados do presente estudo mostraram tendência de declínio da mortalidade por hepatites B e C nos últimos anos no município de São Paulo, particularmente no sexo masculino. Essas infecções foram importantes causas associadas ao carcinoma de células hepáticas e ao HIV. Além disso, destaca-se a proporção de indivíduos que faleceu com menos de 70 anos de idade (cerca de 70%).

A análise de tendência da mortalidade por hepatite B mostrou redução, particularmente em homens. Nos EUA, a tendência da mortalidade por hepatite B entre 1999 e 2007 mostrou que a média anual ajustada por idade foi relativamente constante^19^. Mas em outros locais, como Taiwan, região hiper endêmica em décadas passadas, teve quedas acentuadas^4^^,^^20^^,^^21^. Sugere-se que a diminuição das taxas de mortalidade por hepatite B no Brasil possa ser atribuída em parte à vacinação, com diminuição da prevalência, mas também às medidas de prevenção contra a infecção pelo HIV iniciadas na década de 1980, como observado em outros países^4^^,^^22^^,^^23^.

Em relação à mortalidade por hepatite C, verificou-se tendência de aumento médio de 5,65% ao ano entre 2002 e 2009, em ambos os sexos, e de redução de 4,79% ao ano de 2009 a 2016, no sexo masculino. Nos EUA, a taxa de mortalidade anual padronizada apresentou aumento e ultrapassou a mortalidade por aids, entre 1999 e 2007^19^. No Brasil, chama atenção a queda percentual no segundo período, que em parte pode ser devida ao tratamento instituído no país no início da década anterior, com terapia dupla, utilizada até 2014, incluindo interferon peguilado como um de seus componentes, além do aumento de captação pelos serviços de pessoas infectadas^24^^–^^26^. Em 2015, o Ministério da Saúde instituiu o tratamento com novos antivirais de ação direta, que mostram eficácia de mais de 90% na cura da hepatite C e menores índices de efeitos adversos^27^.

O cuidado clínico para o paciente com HCC tem avançado consideravelmente, devido à evolução dos procedimentos diagnósticos e à melhoria na terapia e prevenção. O tratamento da infecção vem sendo proposto e aperfeiçoado há cerca de 20 anos, com um arsenal terapêutico de eficácia progressivamente melhor.

Em muitos países, a incidência das hepatites B e C diminuiu devido ao menor número de novas infecções. No entanto, a prevalência de doenças hepáticas graves continua aumentando, pois existe um contingente de pessoas infectadas pelo VHB e pelo VHC em décadas passadas que desconhecem sua condição ou que foram diagnosticadas tardiamente, quando apresentaram sinais e sintomas de doença hepática grave, como cirrose e HCC, o que contribui para a morte prematura^9^^,^^28^.

Nos EUA, um estudo de coorte, de 2006 a 2010, mostrou média de idade de 57 anos para os óbitos por hepatite C^29^. Outro estudo conduzido na cidade de Nova York, com casuística de 2000 a 2011, apontou que 64,10% dos óbitos de indivíduos infectados pelo VHC ocorreram antes de 65 anos de idade^30^.

Neste estudo, observou-se que pelo menos dois terços dos óbitos por hepatites B e C acometeram indivíduos com idade inferior a 70 anos, ou seja, os nascidos entre 1945 e 1965 (“*baby boomers*”). No caso da hepatite C, essas pessoas se infectaram principalmente antes da incorporação da triagem em bancos de sangue e de novas tecnologias de produtos e processos relacionados ao controle de sangue e hemoderivados, bem como normas de biossegurança em serviços de saúde^31^. Esse achado fortalece a recomendação para testagem Anti-HCV em pessoas nascidas nesse período, visto que a infecção pode ser assintomática em 75% dos casos^32^, bem como para a instituição do tratamento precoce e a vacinação contra o VHB nesses portadores, conforme protocolo preconizado, impedindo o agravamento do seu quadro^33^.

A significativa proporção de hepatites B e C contribuindo como causas associadas ao HCC e ao HIV é consistente com outros achados na literatura. Uma revisão sistemática de estudos publicados em diversos países mostrou que as hepatites virais crônicas pelo VHB e VHC contribuem para a maioria das ocorrências de HCC^33^. Globalmente, 78% das infecções por HCC foram atribuíveis ao HBV (53%) e HCV (25%), confirmando que as hepatites crônicas B e C representam a principal causa de câncer primário de fígado no mundo^7^^–^^8^^,^^33^. A coinfecção das hepatites virais com o HIV pode ser explicada pelas vias comuns de infecção, principalmente a parenteral e sexual^34^, e está associada ao maior risco de morte^27^^,^^35^. A progressão da fibrose hepática para cirrose é acelerada em indivíduos com hepatite C e HIV concomitantes^36^.

Os dados de mortalidade do presente estudo podem estar subestimados devido à ausência da hepatite B ou C na declaração de óbito, seja como causa básica ou associada^28^. Estudaram-se também as causas associadas de óbito para tentar entender o efeito do sub-registro das hepatites virais B e C como causa básica, principalmente quando associadas à cirrose hepática, HCC e infecção pelo vírus HIV^37^.

As estatísticas de mortalidade segundo causa básica deixam de mostrar o impacto total de uma doença ou agravo, em um conjunto de óbitos^38^. O presente estudo possibilitou esta visão mais ampla e completa, evidenciando a presença das hepatites virais B e C como causas associadas, de forma a contribuir para o conhecimento do perfil epidemiológico desses agravos em nosso meio.

Os dados encontrados mostram a importância da prevenção e do diagnóstico precoce e tratamento das hepatites B e C. A contribuição desse trabalho é fornecer uma linha de base para novos estudos de tendência de mortalidade e de avaliação do impacto de novas intervenções, considerando a ampliação do diagnóstico e da oferta de tratamentos, incluindo os mais recentes antivirais no Brasil, desde 2015^27^, sendo independente do grau de fibrose hepática, desde 2019^39^. Além disso, esses resultados podem subsidiar o alcance de metas previstas para a eliminação das hepatites B e C no município de São Paulo^39^^,^^40^.
